# Lebanon is losing its front line

**DOI:** 10.7189/jogh.11.03052

**Published:** 2021-03-27

**Authors:** Anita Shallal, Chloe Lahoud, Marcus Zervos, Madonna Matar

**Affiliations:** 1 Henry Ford Hospital, Detroit, Michigan, USA; 2 American University of Beirut Medical Center, Beirut, Lebanon; 3 Infectious Diseases, Henry Ford Health System, Detroit, Michigan, USA; 4 Wayne State University School of Medicine, Detroit, Michigan, USA; 5 Infectious Diseases, Notre Dame des Secours University Hospital. Jbeil, Lebanon; 6 The Holy Spirit University of Kaslik, School of Medicine and Medical Sciences, Jbeil, Lebanon

**Figure 1:**
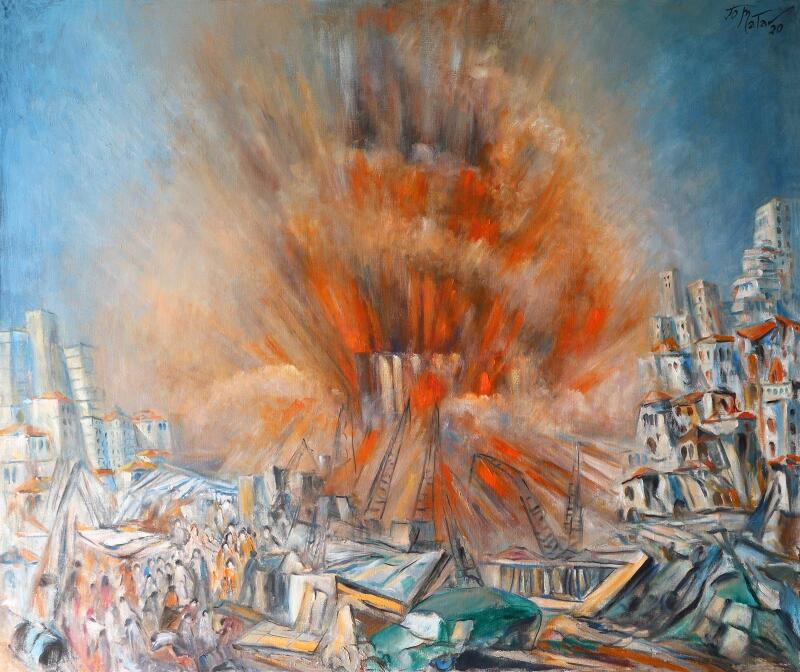
Beirut, the Martyred City (97 × 116.5 cm), painting by Lebanese artist Joseph Matar (reproduced with permission).

Lebanon has been left reeling after an economic recession that had been festering since the end of its civil war in 1990, until its complete collapse in 2019. In the fall of 2019, a time now referred to as the October Revolution, clashes with the government began, and by early 2020, violence was escalating. Then, on 21 February 2020 the first case of COVID-19 in the country was diagnosed. A lack of trust in the government and its ability to handle the pandemic led to protests that continued despite the lockdown and safety measures. The financial crisis worsened as the Lebanese pound lost 81% of its value [1], and the banking systems went completely bankrupt. Amidst this economic and political instability, the health care system continued to face difficulties from a shortage of supplies to an exponential increase in the number of patients, increasing the fragility of the country and leading to an overwhelmed medical system (**Figure 1**).

This was later followed by one of the most powerful non-nuclear explosions in the history of the world on 4 August 2020 in the capital Beirut. The devastating blast left 204 people dead and more than 6500 people injured, in addition to destroying the homes of over 300 000 people and several hospitals [2]. That evening, civilians rushed to the nearest hospitals, centers and streets to help one another without regard for social distancing, given the desperate need for any community response. Since then, Lebanon has seen a substantial spike in COVID-19 cases with over 150 000 reported cases and over 1200 deaths [2]. Later, it was reported that Lebanese officials knew for several years that there were tons of dangerous ammonium nitrate being stored at the port in Beirut. This tragic event reinforced the Lebanese people’s lack of trust in their government. Following public outcry and civil unrest, the Lebanese cabinet resigned, and the Lebanese people were left behind as collateral damage. However, another catastrophe has followed as a result of these events: physicians are leaving to find a better life elsewhere.

Prior to the pandemic and the Beirut bombing, Lebanon was in a fragile state, with a humanitarian crisis of more than one million Syrian refugees. The country is thought to be home to the highest number of refugees per capita in the world [3], placing a tremendous burden on the country’s health care system and health care providers. During the pandemic, the health of refugees deteriorated, as the crowded conditions in tents provided little protection from the virus. In addition, immunization campaigns have been arrested, including delays in vaccinations of preventable illnesses that are already more prevalent in the refugee population, such as the highly contagious measles virus [4].

Issues with fiscal mismanagement has resulted in Lebanon being one of the most indebted countries in the world [5], resulting in dire circumstances for physicians on the front lines. Prior to the pandemic, even the most basic medical supplies were scarce [6], and patients were unable to seek health care when they needed to because they were unable to afford the visit. Even then, some physicians would volunteer at local churches after hours, providing health care to both anti-government protesters and the newly impoverished people alike [7]. After the pandemic struck, the government, short on funds, was unable to set aside a stimulus package for hospitals to help supply much needed resources as the pandemic surged. Some hospitals were left completely dependent on the World Health Organization (WHO), as well as foreign and local non-governmental aid to import essential supplies and equipment, including personal protective equipment (PPE). In addition to the above, the pay for a physician in Lebanon has changed, owing to the enormous devaluation in the country’s currency. It is estimated that the total loss in physicians’ income from the pandemic and devaluation of the Lebanese pound is more than 80% [8]. Those with savings in the country’s banks are unable to retrieve their money. The cost of food, medications, and basic services has tremendously increased. This led to a bigger burden on citizens, as well as health and food insecurity, and fears of impending famine. The headcount poverty rate increased from 28% in 2019 to 55% in 2020, significantly impacting the number of people in Lebanon’s middle class ([9]). In addition, Lebanon’s top universities increased their tuition by 160% [10], leaving students to make a critical decision: to pursue their dreams and sacrifice some of their basic needs due to debt, or to sacrifice their dreams in order to live decently. The downstream, long-term effect of a young population being unable to afford secondary education will not be known for some time.

**Figure:**
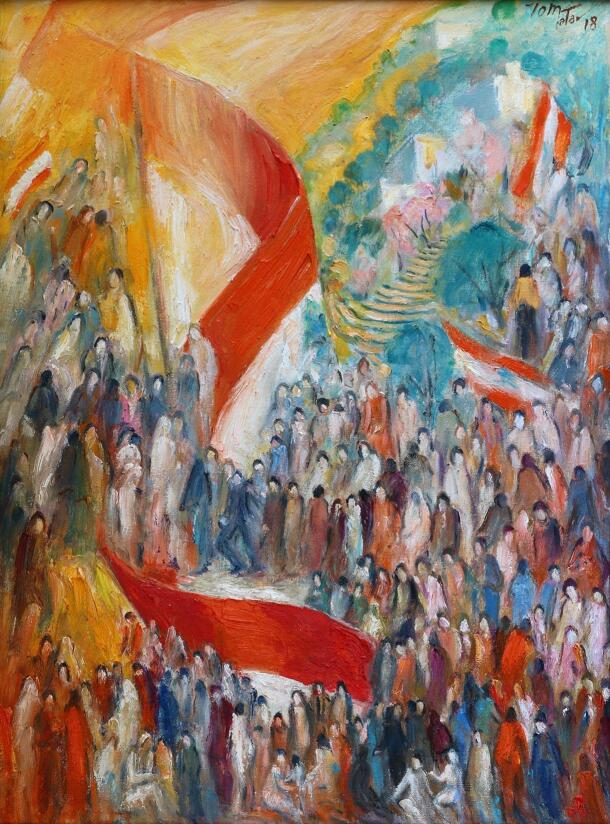
Photo: National Anthem (73 × 54cm), painting by Lebanese artist Joseph Matar (reproduced with permission).

It is estimated that upwards of 400 physicians have already left Lebanon this year, many of them leaving well established university hospitals where they both practiced and taught future physicians. “The absurdity of the explosion and the senseless carnage really shook any little faith I had in the future of Lebanon”, says Dr Faek Jamali, former Professor of General Surgery and Surgical Oncology at the American University of Beirut Medical Center (AUBMC). He left Lebanon in the summer of 2020, moving to the United Arab Emirates in search of stability and safety for himself and his family. Other physicians are planning to leave for Europe or the US, hoping to provide for their family a more secure life. In addition, nurses, who were already in short supply and high demand have also started to leave the country in high numbers [11]. The health care workers who stay face a number of challenges, including their own health and safety when caring for COVID-19 patients [12].

The WHO recommends a bare minimum of 4.45 skilled personnel (which includes physicians, nurses and midwives) per 1000 people in order to deliver safe health care – the term to describe this is known as community health worker density [13]. In Europe and the Americas, this number approaches 14 and 9.6 per 1000 people respectively [13]. A number of countries in the Middle East have failed to meet this minimum requirement [6] including Lebanon, which had under 40 medical staff per 10 000 people in 2018 – prior to the economic collapse of the country. Two years later, this number is unknown. In that same year, Lebanon had one of the highest health expenditures among Arab countries (8.2%) [14]. However, as we have seen in the United States, higher spending does not ensure health care access nor equity, particularly for the poor. As the economic crisis continues, hospitals are laying off and furloughing employees and closing some services – while some hospitals are in danger of closing altogether [8].

We are writing this article to call attention to this crisis within a crisis. The multiple economic strains on Lebanon’s health care system will shortly be followed by thousands of patients without health care providers. We call for continued urgent global support and aid to the country of Lebanon, whether aid be in the form of health or food security and social protection. Additionally, we seek to emphasize the reverberations of damage that can result from a corrupt government, as only a fair and just system can resolve a humanitarian crisis. The economic crisis coupled with a lack of transparency from public officials during the pandemic has resulted in a heavy burden to the already struggling and burned-out frontline. This exodus of physicians is occurring at a time when physicians are needed more than ever. With them is the flight of preventative care, disease prevention, immunization practices, health promotion, and medical education. The full impact of these events on the state and people of Lebanon will affect generations to come.
